# Animal-sourced foods for improved cognitive development

**DOI:** 10.1093/af/vfz039

**Published:** 2019-09-28

**Authors:** Mulubrhan Balehegn, Zeleke Mekuriaw, Laurie Miller, Sarah Mckune, Adegbola T Adesogan

**Affiliations:** 1 Mekelle University Department of Animal, Rangeland and Wildlife Sciences, Addis Ababa, Ethiopia; 2 Feed the Future Innovation Lab for Livestock Systems, Addis Ababa, Ethiopia; 3 International Livestock Research Institute, Addis Ababa, Ethiopia; 4 School of Medicine, Friedman School of Nutrition Science and Policy, and Eliot-Pearson Department of Child Study and Human Development, Tufts University, Boston, MA; 5 Department for Public Health and Health Professions, University of Florida, Gainesville, FL; 6 Department of Animal Sciences, Institute of Food and Agricultural Sciences, University of Florida, Gainesville, FL

**Keywords:** animal-sourced foods, bioavailability, cognitive development, malnutrition

**Implications**

- Animal-sourced foods are the best source of nutrient-rich foods for children aged 6 to 23 mo according to the World Health Organization.
- Studies on the role of animal-sourced foods on cognitive functions are limited, but consistently show compelling benefits.
- Animal-sourced food consumption can positively contribute to school performance in children, lifelong achievement, economic productivity, and social and community outcomes.
- More large-scale randomized controlled longitudinal studies are required to fully understand the link between consumption of animal-sourced foods and cognitive development.
- Improving production of animal-sourced foods does not guarantee increased consumption by children. Complex health, gender, cultural, financial, and religious barriers limit the consumption of animal-sourced food by children, particularly in low- and middle-income countries.
- To increase consumption of animal-source food by vulnerable children, affordability, acceptance, and access must be increased.

## Introduction

Malnutrition continues to be an important problem, despite global increases in food production over the last century. Over 200 million children worldwide fail to meet their developmental potential because of malnutrition and other socio-environmental constraints. In 2018, more than one in five children were stunted and 7.3% suffered from wasting (UNICEF 2019). Although wasting (low weight-for-age) is a measure of acute malnutrition and is associated with mortality, stunting (low height-for-age) is a widely used measure of chronic malnutrition. Children who are stunted in early childhood, particularly in the first 1,000 d, experience reduced growth and physical development and suffer compromised cognitive development leading to lower Intelligence Quotients, worse school performance, greater susceptibility to chronic diseases, increased behavioral problems, and reduced earning potential as adults (De Onis and Branca 2016). In a recent World Bank report, countries where the workforce was stunted in childhood had reduced gross domestic products by about 7% on average globally and 10% to 17% in Africa and South East Asia (Galasso et al., 2016).

Stunting is caused by several interacting factors including poor hygiene and various health-related factors, but the most important cause is an inadequate diet (total calories and essential nutrients) especially during the first 1,000 d. These essential nutrients include iron, zinc, copper, chromium, selenium, iodine, manganese, and molybdenum, and 13 vitamins (vitamin A, vitamin B1, B2, B6 and B12, niacin, folate, pantothenic acid, vitamin C, vitamin D, biotin, vitamin E, and vitamin K). The prevalence of stunting among disadvantaged children in low- and middle-income countries is high and often exceeds 30% in many sub-Saharan and South East Asian countries. This is in part because of the high prevalence of starch-based diets that lack these essential nutrients. Even among the populations that supplement these diets with fruits and vegetables, stunting remains a problem partly because plant foods lack readily bioavailable forms of various micronutrients because they are bound to other compounds like phytate or fiber, markedly reducing their bioavailability (Gibson et al., 2018). Plant foods also completely lack other nutrients, such as vitamin B12, which predisposes people to megaloblastic anemia, developmental delays, failure to thrive, and poor growth in infants and insulin resistance (Williams et al., 2016). Due to widespread consumption of plant-based diets, vitamin B12 deficiency is relatively common, affecting~40% of children and adults in Latin America, ~70% of children in Kenya, and ~80% of children in India (McLean et al., 2007). Vitamin B12 concentrations were deficient in the breastmilk of 89% of 286 nursing Kenyan women indicating that even though breastmilk is the ideal food for infants, its quality and in this case, B12 sufficiency, depends on the adequacy of animal-sourced foods in their diets (Williams et al., 2016).

The concentration of essential micronutrients in plant-based foods is also limited. For example, 2,400 g of spinach contains no more iron than 625 g of beef or 300 g of liver (Gupta 2016). This difference, combined with the greater bioavailability of iron in animal-sourced foods, implies that spinach would be the least favored source of supplying iron to young infants who need dense bioavailable nutrient sources because of the small size of their stomachs. Hence, plant-based foods have limited capacity to fully meet the nutritional needs of infants.

The most deficient micronutrients in human diets globally, iodine, vitamin A, iron, and zinc, are all present in animal-sourced foods. In addition, animal-sourced foods contain greater quantities of high-quality protein, due to their balanced or complete amino acid profile, compared with plant foods. Consequently, animal-sourced foods are ideal for stimulating muscle development and linear growth as well as enhancing cognitive development. These factors outline the vital importance of using animal-sourced foods to prevent or alleviate nutrient deficiencies.

## The Role of Animal-Sourced Foods in Brain Development

There is limited understanding of the specific mechanisms by which animal-sourced foods contribute to improved cognitive development. Studies indicate that bioavailable nutrients in animal-sourced foods such as iron, zinc, iodine, and B vitamins (B12, B6, folate, and riboflavin) enhance cognitive development through their impact on structural brain development via enhancement of myelination, dendritic arborization, and synaptic connectivity (Lövblad et al., 1997). Studies that model brain development using neonatal pigs indicated that early life iron deficiency impairs brain development (Antonides et al., 2015).

Vitamin B12 increases iron and zinc absorption from fiber- and phytate-rich plant staples, contributing to their role in promoting cognitive development (Fairbanks, 1994). Choline and lecithin, found in eggs, are substrates for the synthesis of the neurotransmitter acetylcholine, a chemical known to improve memory (Hasselmo 2006). Animal-sourced foods also contain polyunsaturated fatty acids, such as arachidonic acid and docosahexanoic acid, which together account for about one-fifth of the brain’s dry weight. These fatty acids, along with eicosapentanoic acid, are essential for brain development and function (Bentsen 2017). The accumulation of these fatty acids in the brain is most intense during the third trimester of pregnancy. During the first 2 yr after birth, polyunsaturated fatty acid-dependent processes are involved in expansion of glial cells, neurons, axons, and dendrites and myelination of nerve fibers (Hadley et al., 2016). Furthermore, the high-quality proteins found in animal-sourced foods facilitate specific mechanisms, such as speed of information processing, that are involved in learning tasks such as problem-solving capacity (Neumann et al., 2007). Nevertheless, more research is needed to better understand the biochemical pathways by which consumption of animal-sourced foods positively contributes to cognitive development.

**Figure 1.**
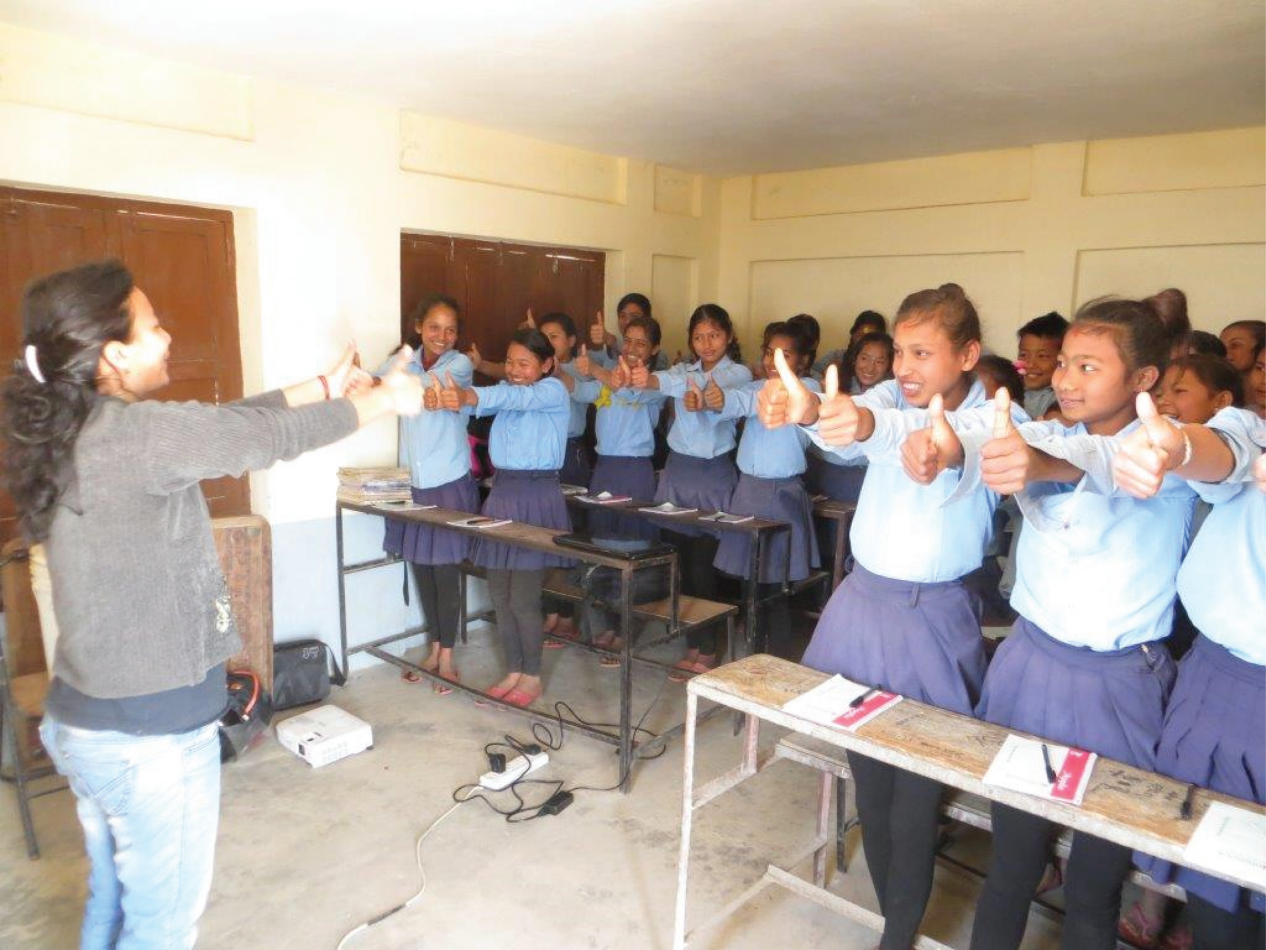
Nepali school children give the thumbs up sign after learning about livestock disease prevention. Photo credit, Renu Shakya.

Animal-sourced foods are the best food-based strategy to prevent stunting and promote cognitive development (Figure 1). Alternatives to animal-sourced food-based provision of the missing nutrients include fortification, biofortification, or supplementation. Fortification and biofortification approaches are important and useful but tend to supply only one of several missing nutrients. Supplementation with missing nutrients is simply not feasible where needed most, such as in the rural areas of low- and middle-income countries due to their unavailability. Supplements would also be prohibitively expensive for the poor. Supplements and fortificants provided as part of research or development interventions do not offer sustainable solutions to these problems in low- and middle-income countries, as these are usually discontinued when donor or government funding ends.

Despite research evidence showing the importance of animal-sourced foods in children’s mental and physical development, diets in poor countries are dominated by starchy grains and legumes that provide an inadequate supply of bioavailable essential micronutrients. Animal-sourced foods constitute only about 5% of the diet in sub-Saharan Africa, compared with about 20% in the United States (Allen 2006). Economic, sociocultural, religious, and other reasons contribute to this lack of consumption. Research results, which clearly demonstrate the role of animal-sourced foods in children’s cognitive development and improvement in adult economic productivity, have unfortunately not been articulated, publicized, and disseminated sufficiently to influence policy and action in agriculture and nutrition (Martorell 2017). Consequently, even when production of animal-sourced food is increased, consumption by children is often not guaranteed due to ignorance about the benefits, cultural taboos against consumption of animal-sourced foods, and their high cost (Mohammed and Aboud 2019).

## Association of Consumption of Animal-Sourced Foods With Cognitive Development or Mental Health: The Evidence

In this paper, via a review of existing evidence from eight intervention and 10 observational studies with data from 61,066 adults and 26,299 children (age range:1–16 yr), across 11 countries, we substantiate the important role of animal-sourced foods in ensuring proper cognitive development of children, and the attendant implications.

Studies that evaluated the effect of inclusion of, or supplementation with, animal-sourced foods with a common or vegetarian-dominated diet on cognition and those that evaluated the association between animal-sourced food diets and cognitive development were included. Studies that did not specifically mention or examine the role of inclusion or supplementation with animal-sourced foods or that examined associations with factors excluding cognitive development were excluded. Databases examined included Google scholar and Science Web. Search terms included various combinations of animal-sourced foods, meat, milk, or eggs with cognition, cognitive development, intelligence quotient, school achievement, or exam scores. The total number of initial references retrieved was 30, of which, 18 were considered pertinent after closer review.

Before discussing the data, it is important to note that there are only a few randomized controlled interventional studies on the impact of animal-sourced foods on cognitive development, perhaps because of the logistical and ethical challenges associated with controlling what human subjects—especially children—consume. Consequently, most of the early evidence comes from animal studies. Available evidence from humans is mostly from cross-sectional or retrospective observational studies that attempt to relate cognitive functions to consumption patterns of animal-sourced food. Such studies are important in terms of developing a hypothesis of association but cannot definitively confirm associations between consumption of animal-sourced foods and cognition, as such studies fail to control for other confounding variables that might influence cognitive development. Over a hundred unique factors have been identified to influence cognitive development in children (Ruiz et al., 2016). Therefore, controlled interventional studies that try to accommodate for other confounding factors such as genetic, environmental, and social factors, are the best for generating robust evidence of association. When observational studies are undertaken, it is almost practically impossible to avoid all other confounding variables that influence cognitive development in children.

Despite the methodological limitations, the few observational and interventional studies that examined the association between consumption of animal-sourced foods and cognitive development identified a clear pattern of positive association. Moreover, many neurological studies involving physical examination and modeling of the human brain suggest the importance of animal-sourced foods or at least the micronutrients that animal-sourced foods supply readily and abundantly on brain development in infants and children.

**Figure 2.**
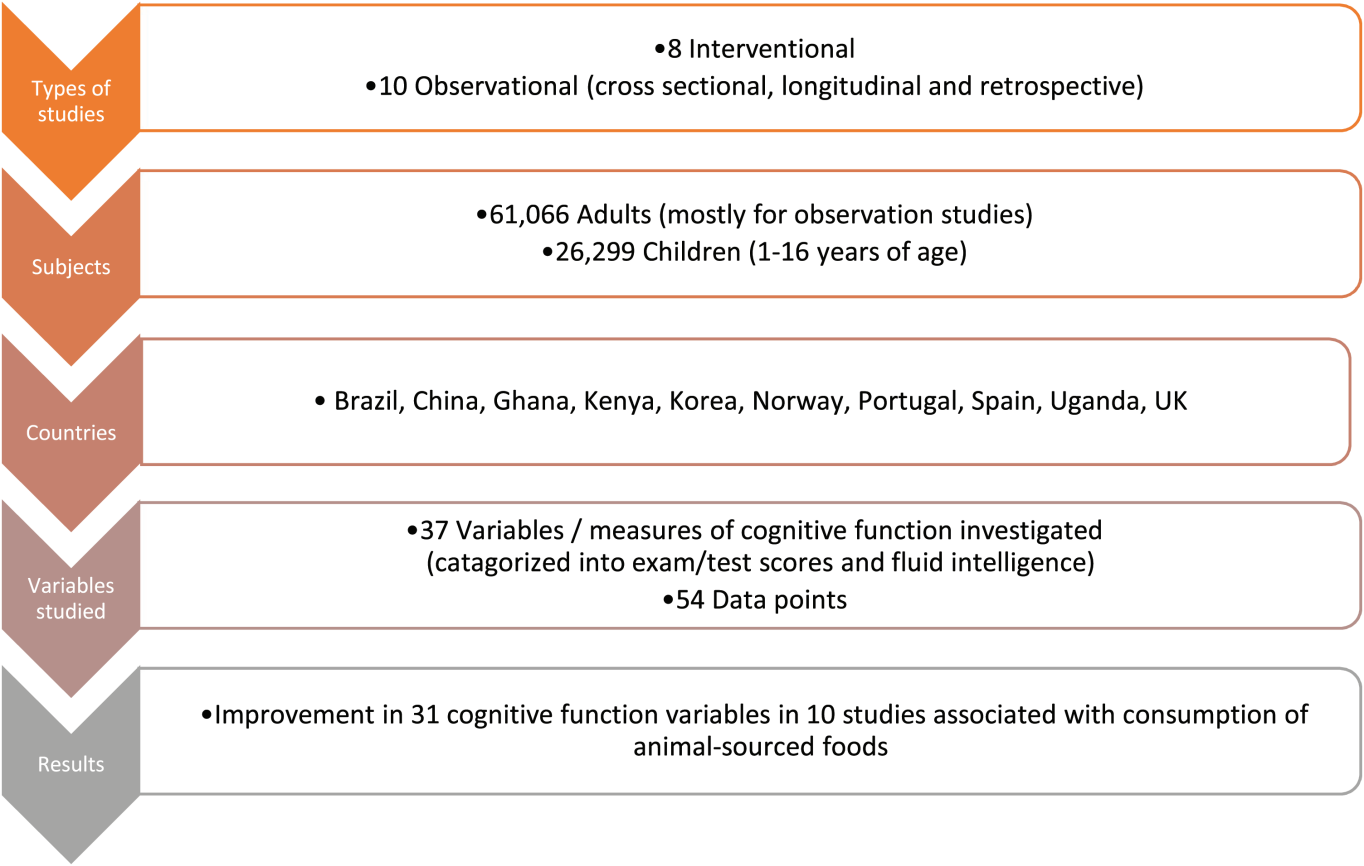
Summary of reviewed evidence of the relationship between consumption of animal-sourced foods and cognitive development.

In this review, studies spanned 12 countries and various age groups, with children dominating the interventional studies and adults in the observational studies. Results consistently show positive associations between consumption of animal-sourced foods and cognitive development. Sources of this evidence are summarized in Figure 2.

## Consumption of Animal-Sourced Foods and Cognitive Development

**Figure 3.**
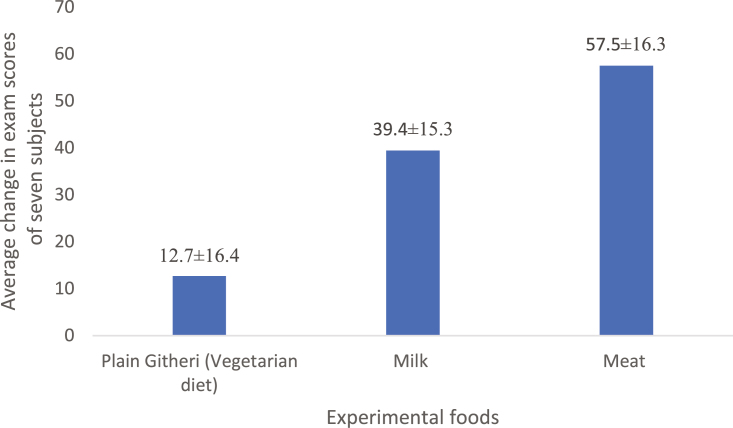
Effect of meat and milk supplementation to a githiri (corn and beans) diet consumed by school children in Kenya on changes in their examination scores averaged across all school subjects and five school terms (Adapted from Hulett et al., 2014).

Malnourished children usually exhibit compromised reasoning, perpetual reduced spatial functioning, poorer school grades, reduced attentiveness, and unresponsive play behavior compared with their well-nourished peers (McLean et al., 2007). A landmark study in Kenya assessed the impact of animal-sourced food supplementation in the diets of school-aged children through a feeding program comparing the impact of supplementation with meat, milk, or energy-equivalent (oil) in the school-based meals of children. This study found that animal-sourced food supplementation (Figure 3) increased exam scores by 45 (meat) and 28 (milk) when averaged across all subjects and school semesters and improved leadership skills and overall behavior of children (Hulett et al., 2014). In the same study, meat supplementation also resulted in improved performance in arithmetic tests, and initiative in leadership (Neumann et al., 2003). Furthermore, recent work demonstrates that consumption of animal-sourced foods by infants and pregnant women is positively associated with better child language, motor, personal, and social skills (Prado et al., 2016).

**Figure 4.**
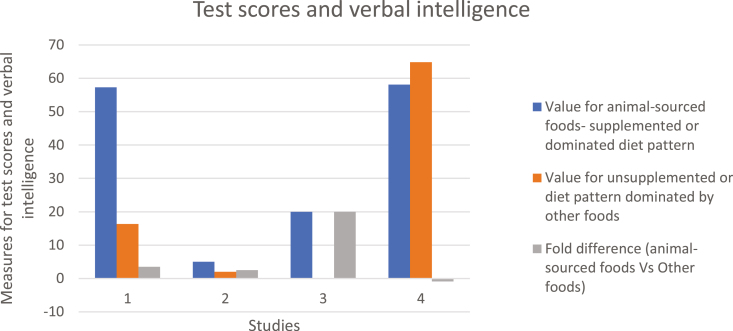
Comparison of test scores and verbal intelligence between subjects supplemented with animal-sourced foods or have animal-sourced foods-dominated diet patterns and subjects supplemented or with diet patterns dominated by other foods. Results are from four studies; 1: (Hulett et al., 2014), 2 and 3: (Neumann et al, 2007), 5: (Mohammed & Aboud, 2019). Nine measures of cognitive development summarized include 1: Combined test scores (Arithmetic, English, Kiembu, Kiswahili, Geography, Sciences and Art); 2: End-of-term arithmetic scores (Raven’s progressive Matrices test); 3: End -of-term total test scores over time (Raven’s Progressive Matrices test); 4: Adjusted mean scores in mathematics and English)

**Figure 5.**
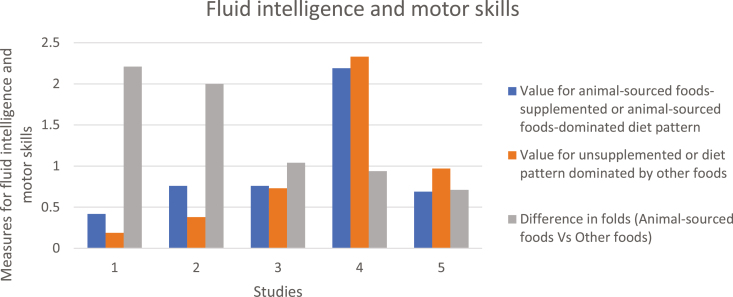
Comparison of Fluid intelligence and motor skills measures between subjects supplemented with animal-sourced foods or have animal-sourced foods-dominated diet patterns and subjects supplemented or with diet patterns dominated by other foods. Results are from five studies 1: (Petrova et al., 2018), 2 and 3: (Heys et al., 2010), 4: (Khee Loo et al., 2017), 5: (Smithers et al., 2012). Five measures of fluid intelligence summarized include 1: Symbol/animal search; 2: 10 word-word recall; 3: 10 word-word recall (adjusted for age, sex and education); 4: Fluid intelligence (Ravens progressive matrices); 5: Intelligence Quotient (IQ)

The various studies we reviewed indicate that supplementation with animal-sourced foods or animal-sourced food-dominated diet patterns increased cognitive functions (test and exam scores) by up to 20 fold and fluid intelligence and verbal skills by up to two-fold (Figures 4 and 5). For instance, children fed vegetarian diets had delays in gross motor development and in speech and language development compared with those fed omnivore diets (Louwman et al., 2000). In another study with elementary school students in Kenya, supplementation with meat resulted in significant improvement in cognitive ability among 555 school children (Black 2003). Supplementation with 8.8 g milk protein per day increased the percentage of correct pattern recognition memory by 5.5% by children when compared with those supplemented with 4.4-g milk protein in a rice-based diet in Ghana (Lee et al., 2018). In a longitudinal study on rural children in Nepal, 43% of the variation in head circumference (an indicator of brain development and cognitive function) was explained by weight for age scores and consumption of animal-sourced foods, with those consuming more animal-sourced foods having greater head circumference scores (Miller et al., 2016).

## Does Timing of Consumption of Animal-Sourced Foods Matter?

Some evidence suggests that the negative effects of malnutrition at certain stages of growth may be irreversible (Levitsky and Strupp 1995) and that the first 1,000 d of life is a critically important window of opportunity for preventing the lifetime consequences of malnutrition. Methodologic constraints make this difficult to directly address. However, only a few studies have examined the impact of consumption of animal-sourced foods in early life on long-term outcomes. For example, improving consumption of animal-sourced foods among children and pregnant women was associated with improved cognitive functions and development in children even later in life (Prado et al., 2016). In the United Kingdom, children whose diets contained meat from 6 to 24 mo were associated with greater intelligence quotient scores at age 8 yr compared with those whose diets contained less meat (Smithers et al., 2012). In a study of more than 20,000 Chinese older adults, limited consumption of meat in childhood (ascertained retrospectively), regardless of animal-source food consumption as adults, was associated with poorer performance on the 10-word recall test (which examines new learning ability and screens for mild cognitive impairment; Heys et al., 2010). There is evidence that the effects of animal-sourced foods on cognitive development may be more pronounced in the long term than in the short term (Hoang et al., 2019). Supplementation with animal-sourced foods in childhood among Guatemalan subjects was associated with improved grade and economic achievement later in life (Maluccio et al., 2009). Cognitive impairment in stunted adults was twice than that of children, implying that the effect of consumption of animal-sourced food on cognitive development may be underestimated in the few existing studies (Hoang et al., 2019).

## Conclusions and Future Perspectives

Studies on the relationship between consumption of animal-sourced food by children, and pregnant and lactating women mostly show improved cognitive development in children and even later in life. This is because animal-sourced food provide the best supply of bioavailable nutrients that are responsible for mental and cognitive development versus vegetarian or starch-based foods. Therefore, for proper cognitive development in children and all the consequent benefits for individuals and communities, children, and pregnant and lactating women, particularly those in low- and middle-income countries, should be provided with proper access to affordable animal-sourced foods.

Although mostly consistent, the evidence on the positive association of animal-sourced food consumption and cognitive development requires further validation. Notably, a few studies, sometimes with mitigating circumstances, found no association or even a negative association between animal-sourced food consumption and certain measures of cognitive development. For instance, soy protein supplementation resulted in greater improvement in nonverbal cognitive (fluid intelligence) in HIV-infected children compared with beef supplementation (Khee Loo et al., 2017). Such discrepancies can be attributed to experimental or other confounding factors, as cognition has been clearly demonstrated to be a result of interaction of a complex set of factors. The relationship between micronutrient deficiency and cognitive and behavioral functions is embedded in a host of other biological and psychosocial risk factors, making consumption of animal-sourced food a necessary, but insufficient, condition for producing cognitive benefits (Ruiz et al., 2016).

The biochemical mechanism by which denser proteins from animal-sourced foods improves cognitive facilities and brain function is not clear, and many studies on the required level of micronutrient supplementation have been inconclusive (Black 2003). Furthermore, there is no empirical evidence on the relative role of different animal-sourced foods such as milk, meat, and eggs on cognitive development and mental health (Neumann et al., 2003), or the optimal daily intakes for different age groups. This information is critical but challenging to acquire because animal-sourced foods improve cognitive function in different ways, making it difficult to establish guidelines (Neumann et al., 2007).

Questions also remain on the relationship between the timing of consumption of animal-sourced foods and long-term impacts on cognition, social development, and depressive symptoms, as most of the evidence is generated from short-term studies. The best way to conclusively establish the required relationships and thereby inform the development of proper dietary guidelines would be through controlled interventional and longitudinal studies that account for confounding and interactive factors. Many more of such studies are urgently needed.

The consistency of evidence on the role of animal-sourced foods on the cognitive development and lifelong achievement of individuals should have important implications on how animal-sourced foods are produced and consumed. Yet, studies on improving human nutrition, so far, have involved little or no discourse with the livestock production sector. Promotion of animal production does not always improve consumption of animal-sourced foods, as many complex factors including high prices, food safety, increased work burden on women, cultural and religious barriers limit the consumption of animal-sourced foods. Strategies and approaches should, therefore, focus on most direct ways for putting affordable animal-sourced foods on the tables of vulnerable families and communities such as smallholder farmers, pastoralists, women headed households, and pregnant and nursing women in low- and middle-income countries (Neumann et al., 2003). Smallholder producers should be supported to improve livestock productivity and efficiency in order to lower production costs and make animal-sourced foods more affordable by the poor. Efforts should also be directed at increasing accessibility to and hygienic preservation of animal-sourced foods for the poor as well as raising awareness about the importance of consumption of animal-sourced foods. The latter should involve highly regarded influencers such as religious leaders, celebrities, or village leaders to overcome cultural, religious, and market limitations. Extension services should specifically focus on educating women, as they are mostly directly involved in preparation, preservation, and serving of food (Neumann et al., 2007). Planned and tailor-made social marketing campaigns and strategic messaging tools should be employed to increase the consumption of animal-sourced foods. Pro-poor marketing and packaging that increase the availability of animal-sourced foods at the poor’s table such as, for example, selling smaller cuts of meat at lower cost would be preferable.

## About the Authors

**Adgebola (Gbola) Adesogan** is Director, Feed the Future Innovation Lab for Livestock Systems and Professor, Ruminant Nutrition at the University of Florida. He is currently the Chair of the Council of Feed the Future Innovation Lab Directors. His research interests include using animal-sourced foods to improve human health and nutrition; improving food and feed safety and quality; and devising sustainable strategies to optimize the performance, health and welfare of livestock. Prior to his tenure at the University of Florida, he was Assistant Professor, Animal Nutrition at the University of Wales, UK. He has earned many honors, including awards from the American Dairy Science Association, the Faculty Commons, and the Florida Agricultural Experiment Station. In 2018, he was named International Educator of the Year by the University of Florida’s College of Agricultural and Life Sciences.

**Mulubrhan Balehegn Gebremikael** is Associate Professor of Livestock Production and Pastoralist Development at Mekelle University, Ethiopia. He holds a PhD in Animal nutrition from the Norwegian Institute of Life Sciences and an MSc in Livestock Production and Pastoralist Development from Mekelle University. His research interests include improving the quality and productivity of animal feeds toward improving the productivity and consumption of animal-sourced foods in developing countries. He is currently working as a research coordinator at the University of Florida. Before his current position, Dr. Mulubrhan was a postdoctoral fellow at the United Nations International Ecosystem Management Partnership and at the Research and Publication Office, Mekelle University.

**Zeleke Mekuriaw** is a Regional Coordinator for the Feed the Future Innovation Lab for Livestock Systems and based at the International Livestock Research Institute (ILRI) in, Addis Ababa, Ethiopia. Since October 2016, he has been coordinating research for development projects in Ethiopia and Rwanda. Previously, he was working for ILRI as a Regional Coordinator and Expert for the Livestock and Irrigation Value Chains for Ethiopian Smallholders project in the Amhara region. Prior to joining ILRI, he was Associate Professor, Applied Livestock Physiology (Reproductive Biotechnology) and Academic and Research Vice President of Debre Tabor University. He was also Dean, College of Agriculture and Environmental Sciences of Bahir Dar University. His research focuses on improving performance of livestock, including camels, sheep and goats, and cattle through improved reproductive performance, genotype, and management.

**Sarah L. McKune** is Assistant Professor in the Department of Environmental and Global Health and the Center for African Studies at the University of Florida. For the past 15 yr, she worked with global health development programs in the West African Sahel, Nepal, Haiti, Uganda, and Ethiopia. She holds a B.A. in French and Sociology, a Master’s in Public Health, a PhD in Interdisciplinary Ecology, and was a postdoctoral Fellow for the CGIAR’s research program on Climate Change, Agriculture, and Food Security. She served as the Director of Public Health Programs for the College of Public Health and Health Professions from 2013 to 2016. She currently serves as the Human Health and Nutrition Cross Cutting Theme leader for the Feed the Future Innovation Lab for Livestock Systems. Dr. McKune’s research seeks to explain the complex, household dynamics that affect child growth and nutritional outcomes, including behavior, women’s empowerment and gender dynamics, climate change, hygiene and sanitation, and livestock ownership.

**Laurie C. Miller** is Professor of Pediatrics, Adjunct Professor of Nutrition and Child Development, Tufts University (Boston, USA). She works with Heifer Nepal as an investigator in several projects supported by the USAID Nutrition Innovation Lab and the Innovation Lab for Livestock Systems. She also serves as a consultant on zoonotic diseases to a World Bank-funded project in Nepal. She is currently a visiting professor and researcher in the Department of Child Psychiatry at Ste. Anne’s Hospital in Paris.
